# High Mortality amongst Adolescents and Adults with Bacterial Meningitis in Sub-Saharan Africa: An Analysis of 715 Cases from Malawi

**DOI:** 10.1371/journal.pone.0069783

**Published:** 2013-07-19

**Authors:** Emma C. Wall, Katharine Cartwright, Matthew Scarborough, Katherine M. Ajdukiewicz, Patrick Goodson, James Mwambene, Eduard E. Zijlstra, Stephen B. Gordon, Neil French, Brian Faragher, Robert S. Heyderman, David G. Lalloo

**Affiliations:** 1 Clinical group, Liverpool School of Tropical Medicine, Liverpool, United Kingdom; 2 Department of Clinical Research, Malawi-Liverpool-Wellcome Trust, Blantyre, Malawi; 3 Department of Microbiology, University Hospitals of Leicestershire NHS Trust, Leicester, United Kingdom; 4 Department of Infectious Disease, John Radcliffe Hospital, Oxford, United Kingdom; 5 Department of Infectious Disease, North Manchester General Hospital, Manchester, United Kingdom; 6 Department of Medicine, College of Medicine Queen Elizabeth Central Hospital, Blantyre, Malawi; 7 Department of Infectious Disease, Rotterdam Centre for Tropical Medicine, Rotterdam, The Netherlands; 8 Institute of Infection and Global Health, University of Liverpool, Liverpool, United Kingdom; Health Protection Agency, United Kingdom

## Abstract

Mortality from bacterial meningitis in African adults is significantly higher than those in better resourced settings and adjunctive therapeutic interventions such as dexamethasone and glycerol have been shown to be ineffective. We conducted a study analysing data from clinical trials of bacterial meningitis in Blantyre, Malawi to investigate the clinical parameters associated with this high mortality.

**Methods:**

We searched for all clinical trials undertaken in Blantyre investigating bacterial meningitis from 1990 to the current time and combined the data from all included trial datasets into one database. We used logistic regression to relate individual clinical parameters to mortality. Adults with community acquired bacterial meningitis were included if the CSF culture isolate was consistent with meningitis or if the CSF white cell count was >100 cells/mm^3^ (>50% neutrophils) in HIV negative participants and >5 cells/mm^3^ in HIV positive participants. Outcome was measured by mortality at discharge from hospital (after 10 days of antibiotic therapy) and community follow up (day 40).

**Results:**

Seven hundred and fifteen episodes of bacterial meningitis were evaluated. The mortality rate was 45% at day 10 and 54% at day 40. The most common pathogens were *S.pneumoniae* (84% of positive CSF isolates) and *N.meningitidis* (4%). 607/694 (87%) participants tested were HIV antibody positive. Treatment delays within the hospital system were marked. The median presenting GCS was 12/15, 17% had GCS<8 and 44.9% had a seizure during the illness. Coma, seizures, tachycardia and anaemia were all significantly associated with mortality on multivariate analysis. HIV status and pneumococcal culture positivity in the CSF were not associated with mortality. Adults with community acquired bacterial meningitis in Malawi present with a severe clinical phenotype. Predictors of high mortality are different to those seen in Western settings. Optimising in-hospital care and minimising treatment delays presents an opportunity to improve outcomes considerably.

## Introduction

Since the beginning of the HIV epidemic, many high prevalence Sub-Saharan African countries have reported an increase in patients presenting to health care facilities with acute bacterial meningitis (ABM) [1], [2]. Following the introduction of penicillin in the 1950s mortality rates from ABM in resource rich settings have improved from 45–50% to 11–25% in the last decade [3]–[7]. This improvement is associated with early institution of antibiotic therapy and better supportive care [4], [8]. In contrast, adult ABM mortality rates in sub-Saharan Africa have been reported to vary between 54–70% without any change over time [9]–[12] and survivors experience significantly higher rates of disabling neurological sequelae compared to European patients [13].

Interventions such as adjunctive dexamethasone and glycerol have failed to impact on this high mortality in large randomised controlled trials in Malawi and thus ABM remains a considerable clinical challenge [14], [15]. In European and American cohorts important risk factors for poor outcome including advanced age, acute hyperglycaemia and immunosuppression have been identified [16], [17], however data are lacking from resource-poor settings. We took advantage of the large body of prospective clinical trial and observational data collected in Blantyre, Malawi to investigate these dramatically high adult ABM mortality rates and identify risk factors for a poor prognosis.

## Methods

### Setting

Queen Elizabeth Central Hospital (QECH), Blantyre is a 1250-bed government funded hospital with free care at the point of delivery that admits 50,000 patients per year. QECH serves a population of approximately 1 million including the city of Blantyre. The diagnostic laboratory at the Malawi Liverpool Wellcome Trust Clinical Research Programme (http://www.mlw.medcol.mw) has provided a quality controlled cerebral spinal fluid (CSF) and blood culture service for QECH for more than 10 years.

### Study Selection

We identified all clinical trials and observational studies (published and unpublished) undertaken at the Department of Medicine, College of Medicine, QECH between 1990-to the current time, where adults with ABM were recruited prospectively and data were freely available. Database access for each study was obtained from the principal investigator.

### Inclusion and Exclusion Criteria

Included participants were over 13 years of age (adult ward admission age) and enrolled in a clinical study of ABM at QECH with either CSF proven microbiological evidence of ABM, or a high clinical index of suspicion of ABM plus a CSF white cell count that was >50% neutrophils and >100 cells/mm^3^ in HIV negative or 5 cells/mm^3^ in HIV positive. The latter was selected because the CSF inflammatory response in ABM may be attenuated in HIV infected African adults [18]. Participants were excluded if the CSF microscopy or culture was positive for *Cryptococcus neoformans* or *Mycobacterium tuberculosis*, if the CSF white cells were >50% lymphocytes, or on study specific criteria, such as type two diabetes for the glycerol trial [11].

### Outcome Analysis

The outcome measure for included subjects was mortality which was measured at day 10 and at day 40. Day 40 was prospectively agreed as the last follow up time point in included studies. Day 0 was recorded as the date and time of entry into the hospital; day 1 was 24 hours from this point.

Variables were standardised and a single database was created. The first recorded clinical observations on admission were used. Eighteen clinical and demographic parameters were selected on the basis of previous published associations and suspected risk factors, and subjected to analysis for associations with death [16], [17], [19]. Age >40 was selected as the current life expectancy in Malawi is 45–50 years. Glycerol receipt was included to account for the excess mortality seen in that trial [13]. The analysis plan was designed first to test all 18 variables against mortality using univariate analysis and enter these variables into a multivariate model to test the strength of any associations seen. Clinically relevant groupings for continuous variables were created for the multivariate model to aid interpretation of the results. We have reported the seasonal nature of invasive pneumococcal disease in Malawi, and used this data to investigate if mortality was also seasonal [9], [20].

### Ethical Approval

All constituent studies had been approved by the College of Medicine Research and Ethics Committee in Malawi, and by the Liverpool School of Tropical Medicine Research Ethics Committee and conformed to institutional guidelines. All participants in the included clinical trials and studies gave written informed consent, or this was given by a named guardian if the participant was under 18 years of age.

### Statistical Methods

Data were analysed using SPSS/PASW version 20. Univariate analysis was performed for each variable. For continuous variables, parametric (Student t) and non-parametric (Mann-Whitney U) tests were used to compare survivors and non-survivors; Fisher exact/chi-square tests were used to compare categorical variables. A multivariate analysis model was generated using backwards stepwise logistic regression methods to estimate the influence of each variable on clinical outcome. Each model was compared at each step with the previous model and only variables with on-going significance remained in the analysis. Data from the final model retaining all significant variables were reported. The strength of relationships was expressed using odds ratios with 95% confidence intervals. All statistical tests were two-tailed, and a p value of <0.05 was used to denote statistical significance. Due to the heterogeneous nature of the studies included, some significant variables had missing data; if >50% of the data were missing, these variables were excluded from the multivariate analysis. The multivariate analyses were repeated using multiple imputation methods to enable subjects with missing data to be included; this did not alter the results and these analyses are not presented.

## Results

### Study and Patient Selection

Five studies were identified. One observational study was excluded as it was conducted prior to the introduction of ceftriaxone in 2001 under different admission conditions [9]. One vaccine trial was excluded as specific clinical data for the patients with meningitis were not available [21]. The three included studies were two clinical trials, testing dexamethasone [11] or glycerol [15] adjunct therapy and an unpublished prospective cohort study investigating risk factors for mortality (Table 1). All studies recruited patients over 13–16 years with ABM and used ceftriaxone as the first line antibiotic. A total of 891 participants were identified. We excluded 115 patients who had either proven fungal (n = 52) or tuberculous meningitis (TBM) (n = 25), or a CSF white cell count of <5 cells/mm^3^ (n = 38). A further sixty one patients were excluded because no mortality data were available at day 10 yielding 715 cases for the analyses. We excluded a relatively high proportion of participants from the cohort study (118/161) compared to 26 from the glycerol trial and 32 from the dexamethasone trial. This was due to a high number of patients in this database either not meeting the white cell count inclusion criteria for this analysis or having mortality data available. Free access to HIV treatment become routinely available during the study period; co-trimoxazole prophylaxis in 2002 and anti-retroviral therapy (ART) from 2004 [20].

**Table 1:** Details of included studies.

Study name	Study dates	Type of study	Intervention tested	Total number of cases(number included indatabase)	Total number ofdeaths (%)
**Steroids in Adult Meningitis study** **(SAM)** [**^11^**]	2001–2004	Randomised Controlled Trial	Intravenous Dexamethasone adjunct to antibiotics	465 (433)	233 (54)
**Glycerol in Adult** **Meningitis (GLAM)** [**^15^**]	2006–2008	Randomised Controlled Trial	Oral glycerol adjunct to antibiotics	265 (239)	129 (54)
**Cohort observational** **study (unpublished)**	2009	Observational cohort	No intervention	161 (43)	22 (51.1*)

* this study only collected data to day 10 hospital discharge and not day 40 follow up.

### Clinical and Laboratory Findings

The median age was 31 years (inter-quartile range IQR 25–38) and half were female (50.3%). 20 participants (2.8%) were adolescents. 608/695 (87%) of those tested were HIV antibody positive, of which 36 (5.9%) were on ART (Table 2). CD4 cell counts were not routinely measured during the time period studied.

**Table 2:** Demographics/characteristics of study patients.

Characteristic on presentation	Number of observations available	Value (% or Inter Quartile Range IQR)
Clinical observations		
Deaths at day 10	715	324 (45.3)
Total deaths at 40 day follow up	668	363 (54.3)
Female sex	715	364 (50.3)
HIV positive	694	607 (87)
Antiretroviral therapy	607	35 (5.7)
Out of hours admission	655	315 (48)
Median age	715	31 (25–38)
Headache or history of acute headache	691	683(99)
Neck stiffness	715	536 (75)
Photophobia	683	106 (15.5)
Median Glasgow Coma Score recorded	649	12.0 (9–14)
GCS>8–<11 (significant alteration of mental status)	649	169(26)
GCS<8 (coma)	649	113 (17.4)
Recorded acute seizure	714	319 (44.9)
Seizure post discharge	416	89 (21)
Median mean arterial blood pressure (mmHg)recorded	593	86 (79.9–96.7)
Median pulse (bpm)	596	100 (88–116)
Mean temperature (°C) recorded	668	38.1 (37.2–39)
Median recorded oxygen saturations	207	96% (93–97)
Microbiology		
Positive CSF culture	712	419 (58.8); *S.pneumoniae* n = 356 (84.9); *N.meningitidis* n = 17 (4.1); *H.influenzae* n = 3 (0.7); Other n = 43 (10.2)
Median CSF white cell count (cells/mm^3^)	707	480 (170–1680)
Positive blood culture	660	185 (27.8%); *S.pneumoniae* n = 126 (68.4); *N.meningitidis* n = 8 (4.1); *H.influenzae* n = 1 (0.4); Enteric Gram negative n = 27 (14.9); Other n = 23 (12.4)
Blood tests		Median (IQR)
Sodium (mmol/L)	43	135 (130.9–139.1)
Haemoglobin (g/dL)	643	10.8 (9.0–12.7)
Glucose (mmol/L)	546	6.7 (6.0–98.7)
Creatinine (mg/dL)	40	1.1 (0.9–1.2)

The overall mortality rate was 45.3% in hospital by day 10, and 54.3% by day 40, with no significant difference across the three studies (Table 2). Altered mental status and coma were common. The median presenting Glasgow Coma Score (GCS) was 12 (IQR 9–14.0) and 113 (17.4%) of participants presented with a GCS of 8 or less. 319 (44.6%) participants experienced an acute seizure and 89 participants experienced seizures after discharge. Fever >38°C (88.5%), headache or history of headache (99%) and neck stiffness (75%) were common, however photophobia was rare (15.5%). Septic shock was uncommon; the median pulse rate was 100 beats per minute (IQR 88–116) and the median mean arterial pressure (MAP) was 86 mmHg (IQR 79.9–96.7). Neurological disability (with the exception of post discharge seizures) and hearing loss were reported differently across the included studies and could not be sufficiently standardised for inclusion on the analysis.

CSF culture was positive in 419 of 712 specimens (59%). *Streptococcus pneumoniae* was the most common isolate (356/419, 85%) followed by *Neisseria meningitidis* (17/419, 4%) (Table 2). Other isolates included Non-typhoidal Salmonellae (NTS), *Escherichia coli*, Group A streptococci and *Haemophilus influenzae.* The median CSF white cell count was 480 cells/mm^3^ (IQR 170–1680). CSF opening pressure was not routinely recorded. Blood cultures were positive in 185/664 (28%) of individuals; the most commonly isolated organisms being *S.pneumoniae* (126/185, 68%), enteric gram negative organisms (27/185, 15%) and *N.meningitidis* (8/185, 4%). Of 352 pneumococcal CSF isolates where paired blood culture data were available, 139 (40%) were positive. Comprehensive data on antimicrobial resistance were not available. Pneumococcal serotype was determined in 128 of 352 isolates; 56 (43.8%) were serotype 1. Other serogroups were 6 (4.7%), 7 (3.1%), 9 (4.7%), 12 (3.9%), 14 (4.7%), and 19 (3.1%). Mortality was 59% (33/56) for the participants infected with serotype 1 and 50.1% (36/71) for the other serogroups. Available data were too few to analyse this trend further.

### Delays to Presentation to Hospital and Clinical Care

Presentation out of routine working hours comprised 318 (44.5%) of admissions. For a subset of patients with culture proven ABM ‘the observational cohort’, (n = 26) data were collected prospectively relating to the patient pathway on a standard form to investigate delays in presentation, investigation and initiation of therapy. Nine of 26 patients (35%) died before day 10. Median self-reported journey time to QECH was 0.8 hours (IQR 0.5–1.6) in non-survivors, and 1.5 hours (IQR 0.7–2.0) in survivors. Median time from presentation to medical review was 2.8 hours (IQR 0.8–5.8) in non-survivors and 1 hour (IQR 0.6–3.0) in survivors. The median time from presentation at the hospital with meningitis to receipt of intravenous antibiotics was 5.0 hours (IQR 2.5–8.9) in non-survivors and 2.7 hours (IQR 1.7–6.0) in survivors. No time difference reached statistical significance.

### Prognostic Clinical Features

Eighteen parameters were subjected to univariate analysis for associations with survival at day 10 (n = 715) and day 40 (n = 668). There was no statistically significant difference between these two time points for any of the variables except pneumococcal culture and therefore the day 40 follow up data are presented (Table 3).

**Table 3:** Predictors of mortality at day 40 on univariate and multivariate analysis.

Parameter			Day 40		Univariate (unadjusted)		Multivariate (adjusted)**	
			Alive	Dead	Odds ratio (95% CI)	p	Odds ratio (95% CI)	p
**Sample size**			304	363				
**Age**		**mean (sd)**	31.5 (10.7)	34.2 (11.4)	1.023 (1.009∶ 1.038)‡	0.002		
		**≤40 years**	256	285 (52.7%)	–	–	–	–
		**>40 years**	48	78 (61.9%)	1.460 (0.981∶ 2.171)	0.062	1.327 (0.858∶ 2.055)	0.204
**Gender**		**male**	149	177 (54.3%)	–	–	–	–
		**female**	155	186 (54.5%)	1.010 (0.754∶ 1.370)	0.948	0.894 (0.621∶ 1.287)	0.548
**HIV status**		**negative**	43	37 (46.2%)	–	–	–	–
		**positive**	254	316 (55.4%)	1.446 (0.904∶ 1.370)	0.124	1.184 (0.700∶ 2.001)	0.528
		**not known**	7	10				
**Out of hours admission**		**no**	138	173 (55.6%)	–	–	–	–
		**yes**	134	162 (54.7%)	0.964 (0.700∶ 1.328)	0.824	0.949 (0.661∶ 1.362)	0.777
		**not known**	32	28				
**GCS**		**mean (sd)**	12.2 (2.8)	10.2 (3.6)	0.825 (0.786∶ 0.867)‡	<0.001		
		**≥11**	205	168 (45.0%)	–	–	–	–
		**8–11**	69	94 (57.7%)	1.662 (1.146∶ 2.411)	0.007	1.665 (1.086∶ 2.554)	0.007
		**<8**	20	84 (80.8%)	5.125 (3.021∶ 8.695)	<0.001	5.999 (3.314∶ 10.861)	<0.001
		**not known**	10	17				
**1 or more acute**		**no**	216	207 (48.9%)	–	–	–	–
**seizure episodes**		**yes**	87	156 (64.2%)	1.871 (1.353∶ 2.588)	<0.001	1.552 (1.075∶ 2.241)	0.019
		**not known**	1	0				
**Seizure post-discharge**		**no**	271	57 (17.4%)	–	–		
		**yes**	23	64 (73.6%)	13.230 (7.591∶ 23.057)	<0.001	†	†
		**not known**	10	242				
**SpO_2_**		**mean (sd)**	95.2 (2.7)	93.5 (5.7)	0.888 (0.806∶ 0.978)‡	0.016	†	†
		**>95**	52	48 (48.0%)	–	–		
		**92–95**	26	32 (55.2%)	1.333 (0.696∶ 2.552)	0.385	†	†
		**<92**	11	29 (72.5%)	2.856 (1.287∶ 6.339)	0.010		
		**not known**	215	254				
**Pulse rate**		**mean (sd)**	96.9 (18.6)	103.1 (19.0)	1.018 (1.008∶ 1.027)‡	<0.001		
		**<100**	172	162 (48.5%)	–	–	–	–
		**100–120**	39	57 (59.4%)	1.552 (0.979∶ 2.459)	0.061	1.422 (0.844∶ 2.397)	0.186
		**>120**	44	76 (63.3%)	1.834 (1.194∶ 2.816)	0.006	1.800 (1.084∶ 2.987)	0.023
		**not known**	49	68				
**MAP (mmHg)**		**mean (sd)**	89.7 (13.0)	89.0 (15.5)	0.996 (0.985∶ 1.008)‡	0.530		
		**<90**	152	174 (53.4%)	–	–	–	–
		**90–100**	57	66 (53.7%)	1.011 (0.667∶ 1.533)	0.957	0.972 (0.622∶ 1.520)	0.902
		**>100**	44	56 (56.0%)	1.112 (0.708∶ 1.745)	0.645	1.107 (0.636∶ 1.925)	0.720
		**not known**	51	67				
**Respiratory rate**		**mean (sd)**	23.8 (9.6)	24.2 (6.3)	1.007 (0.954∶ 1.064)‡	0.790	†	†
		**not known**	246	299				
**Anuria**		**no**	208	252 (54.8%)	–	–	–	–
		**not known**	96	111				
**CSF culture positive for** ***S.pneumoniae***		**no**	145	180 (55.4%)	–	–	–	–
		**yes**	158	181 (53.4%)	0.923 (0.680∶ 1.253)	0.606	0.704 (0.488∶ 1.016)	0.061
		**not known**	1	2				
**Plasma glucose**		**mean (sd)**	7.3 (2.7)	7.4 (3.8)	1.007 (0.957∶ 1.059)‡	0.793		
**(mmol/L)**		**≥9**	46	45 (49.5%)	–	–	–	–
		**6–9**	90	102 (53.1%)	1.159 (0.703∶ 1.909)	0.563	1.292 (0.748∶ 2.234)	0.358
		**≤6**	101	123 (54.9%)	1.245 (0.764∶ 2.028)	0.379	1.423 (0.763∶ 2.653)	0.268
		**not known**	67	93				
**Haemoglobin (g/dL)**		**mean (sd)**	11.4 (2.9)	10.5 (2.9)	0.893 (0.841∶ 0.949)‡	<0.001		
		**>14**	50	32 (39.0%)	–	–	–	–
		**11–14**	95	101 (51.5%)	1.661 (0.983∶ 2.807)	0.058	1.836 (1.006∶ 3.352)	0.048
		**8–11**	104	134 (56.3%)	2.013 (1.206∶ 3.360)	0.007	2.598 (1.422∶ 4.747)	0.002
		**5–8**	26	45 (63.4%)	2.704 (1.404∶ 5.210)	0.003	3.404 (1.603∶ 7.229)	0.001
		**<5**	4	11 (73.3%)	4.297 (1.259∶ 14.662)	0.020	6.343 (1.861∶ 21.624)	0.003
		**not known**	67	93				
**Glycerol treatment**	**no**		58	53 (47.7%)	–	–	–	–
	**yes**		47	76 (61.8%)	1.770 (1.051∶ 2.978)	0.032	1.884 (1.092∶ 3.249)	0.023
	**not known**		199	93				
**Na (mmol/L)** *	**median (range)**		137 (125∶199)	132 (115∶152)	0.928 (0.834∶ 1.032)‡	0.166	†	†
		**not known**	283	341				
**Creatinine (mg/dL)** *	**median lpar;range)**		1.05 (0.7∶1.5)	1.15 (0.5∶10.8)	4.441 (0.682∶ 28.925)‡	0.119	†	†
		**not known**	286	341				

† proportion of missing data >50% so variable excluded from multivariate analysis.

‡ odds ratio for a unit increase in predictor variable.

* values at day 10 presented as too few observations available at day 40 data for a meaningful analysis.

** 4 cases excluded due to missing observations.

The following clinical parameters were significantly associated with poor outcome on univariate analysis: age, clinical presentation with altered mental status, acute and post discharge seizures, hypoxaemia, tachycardia, hyponatraemia, anaemia and treatment with glycerol. Neither gender, out of hours admission, HIV antibody status, blood glucose, mean arterial blood pressure, anuria, respiratory rate, nor serum creatinine were associated with poor outcome (Table 3). Of the 339 participants with *S.pneumoniae* isolated from the CSF, 181 (54%) died and 158 (46%) were alive at day 40 OR 0.923 (0.68∶ 1.25) p = 0.61. As culture of *S.pneumoniae* in the CSF and HIV status have been associated with mortality in other studies of bacterial meningitis [22], [23] these data were added to the multivariate model with the other tested variables. Coma scores were grouped into three categories: <8, 8–11 and >11. Oxygen saturations, post discharge seizures, respiratory rate, anuria, creatinine and sodium had >50% of data missing and were excluded. Seizures were divided into acute (pre-hospital and in hospital) and seizures during follow up. Haemoglobin and pulse rate were divided into clinically significant groupings.

Presentation with coma was the strongest independent predictor of outcome after multivariate analysis; OR for death with a GCS<8/15 was 5.9 (95% CI 3.31∶ 10.86) p<0.001, GCS of 8–11 was 1.665 (1.08∶ 2.5 ) p = 0.007. Acute seizures were associated with death, OR 1.55 (1.08∶ 2.24) p = 0.019. The severity of anaemia was associated with a corresponding increasing risk of mortality, OR death of haemoglobin (Hb) 5–8 g/dL was 3.41 (1.61∶ 7.23) p = 0.001; at Hb<5 g/dL the OR was 6.34 (1.86∶ 21.62) p = 0.003. Every 1 g/dL increase in haemoglobin was associated with a 12% reduction in mortality, OR 0·88 (0.82–0.94). Tachycardia >120 bpm was also associated with death, OR 1.80 (1.08∶ 2.99) p = 0.023. Glycerol receipt was independently associated with mortality 1.88 (1.09∶ 3.25) p = 0.023. Age >40 years was not significant, OR 1.33 (0.86∶ 2.06) p = 0.20.

Neither CSF pneumococcal culture positivity nor HIV status were significantly associated with mortality at day 40. The OR of death were 0.70 (0.49∶ 1.02) p = 0.06 for pneumococcal infection, and 1.18 (0.70∶ 2.00) p = 0.52 for HIV infection. No parameter reaching non-significance at the univariate level was shown to have significant association at the multivariate level.

**Figure 1:**
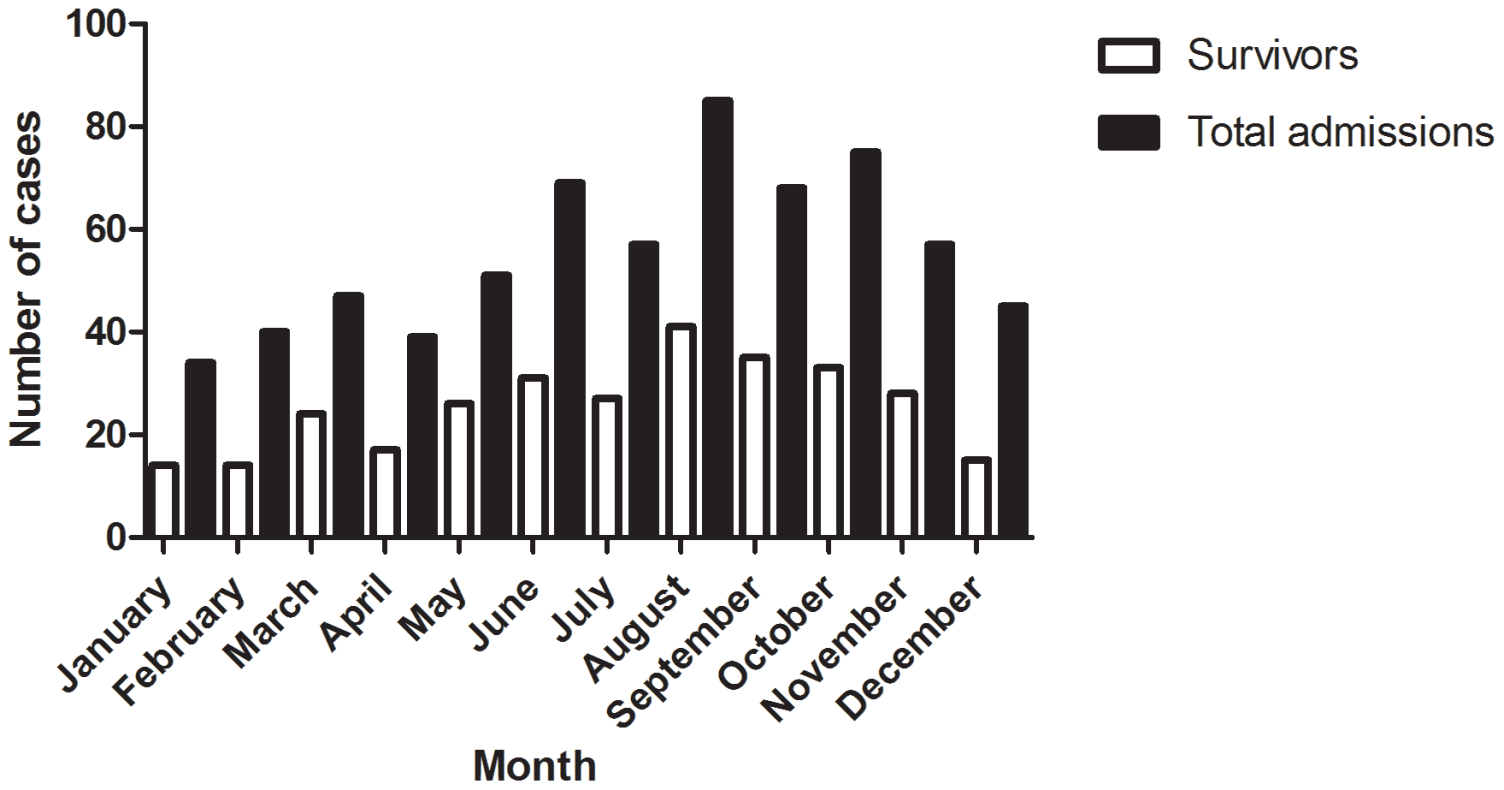
Cases and mortality by month of admission at day 40 follow up.

Mortality was not seasonal; 279 (36%) of cases presented in the cold winter season (May-September), with 145 (52%) deaths and 388 (64%) cases presented during the remaining hot months of the year with 217 (56%) deaths p = 0.34 (Figure 1).

## Discussion

Adults and adolescents presenting to hospital in Malawi with bacterial meningitis are young, largely HIV positive and have a very high mortality rate, contrasting significantly with published data from high income countries [3], [24]. Eighty seven percent of participants in our study were HIV antibody positive compared to the QECH medical inpatient rate of 76% and a current adult national population prevalence of 12% [25]. These figures are similar to data from meningitis patients in other African centres [12], [18], [26]; invasive infection with *Streptococcus pneumoniae* is more common in HIV infected than HIV uninfected individuals in sub-Saharan Africa [21], [27]. HIV infected adults in our setting have higher rates of pneumococcal carriage than HIV uninfected individuals [Kamng’ona et al 2013 unpublished]. The high rates of bacterial meningitis in HIV positive adults may be potentially related to increased susceptibility to *S.pneumoniae* infection via altered mucosal and cell-mediated immunity [28]. Immunocompromise has been associated with ABM in adults, but HIV has not to date been proven to be an independent risk factor [9], [23], [24], [29]. In contrast to the apparently increased frequency of disease, HIV sero-positivity was not significantly associated with mortality. However the numbers of HIV negative patients were small, with an additional very small number where no sero-status was known. All the HIV antibody positive participants in this study were WHO clinical stage 3 or 4, classified as such by the diagnosis of bacterial meningitis as stage 3, becoming stage 4 with one prior documented invasive bacterial infection. In children in sub-Saharan Africa HIV may be a risk factor for death from ABM, [22], [26], [30] but data in African adults from other centres are lacking. Although epidemiological data are scarce, preliminary culture positive ABM incidence estimates for 2010 are 12.6 cases of ABM per 100 000 adult population in Malawi [Wall, Everett, et al 2013 unpublished] compared to 1.38–3.2/100 000 adults in high income countries [16], [23], [24].

A striking finding from this very large ABM dataset was the excessive mortality which is much higher than in series from industrialised countries and has remained unchanged for 10 years [9], [16], [23], [24]. This is in direct contrast with a large study of ABM in Holland (n = 696), where patients presented early and the overall mortality was 21%. In that cohort over 50% of patients presented within 24 hours of symptom onset. 14% of participants in Holland vs. 17% in Malawi presented in coma (GCS<8/15), 5% vs. 44.9% had a seizure during the illness and 20% vs. 22% presented with a tachycardia >120 bpm [16]. The studies included in this analysis all had broad inclusion criteria and recruited patients admitted with ABM at night and weekends, and the included cases were representative of all adults with ABM presenting to our centre, the risk of selection bias was low. Some cases in the studies were missed due to early mortality, this was predominately seen in the glycerol study, but as clinical data were not available for these participants, we were not able to include these cases in the analysis. Furthermore the glycerol study was stopped early by the study safety monitors due to higher than expected mortality in the glycerol group. The analysis was re-run without data from participants who received glycerol, no significant differences were found. Data from these participants were included in the database as the presenting characteristics were equally matched with the other studies and glycerol is shown to be an independent predictor of mortality.

Early access to resuscitation and antibiotic treatment has been instrumental in reducing mortality rates from paediatric meningitis [4], [31]–[34]. Our limited analysis of the patient journey suggests delays in clinical management may be important. The median five hour delay before non-survivors received antibiotics may have contributed to poor survival as shown in other series, although our data are small [4], [8]. These delays were predominately due to staff resource constraints and the use of a complex triage system. Delay to prompt medical review, acute resuscitation and suboptimal intensive care support may also have contributed. Preliminary data from our centre suggests that pre-hospital delays in seeking treatment for meningitis stem from a lack of understanding and recognition of the serious nature of the illness, cultural inhibitions and long, expensive journeys to hospital [Desmond 2013 PLOS ONE in press]. The self-reported journey times recorded must be interpreted with caution as few patients owned a watch or mobile phone.

Similarly to the Dutch cohort, we show that coma and tachycardia are independently associated with death on multivariate analysis [16]. In addition we show acute seizures, glycerol administration and anaemia are independent predictors of mortality in Africa. Anaemia is extremely common in this setting and may generally be associated with a poor clinical outcome in hospital [11], [35], [36], however its independent association with poor outcome from meningitis warrants further investigation.

*S.pneumoniae* was the most common organism isolated from CSF. Only 4% of cases were due to *N. meningitidis*, all of whom survived. Malawi is south of the African meningitis belt, and meningococcal epidemics occurring during the study period did not affect Malawi. Our results therefore are not generalizable to that region, but are applicable to other African countries in sub-Saharan Africa with high rates of HIV infection and invasive pneumococcal disease. Mortality was highest in those with *S.pneumoniae* serotype 1 [14]. Serotype 1, is included in the 13 valent pneumococcal conjugate vaccine introduced in Malawi in 2011, and surveillance of the impact of vaccination on invasive pneumococcal disease is likely to be important [37].

In contrast to Western data, a sub-analysis (not presented) did not show an association between *S.pneumoniae* culture positivity and poor neurological outcome or seizures in survivors [16].

All studies used ceftriaxone as the first line antibiotic. Only a very small proportion were taking antiretroviral therapy (ART) and we do not have data on co-trimoxazole use. We reported pneumococcal resistance between 2000–2009 to the following antibiotics: multi-drug resistance (co-trimoxazole, tetracycline and chloramphenicol) in 30%, penicillin in 15%, ceftriaxone in 0%; co-trimoxazole resistance increased from 74% to 93% [20] [38] ^.^ Our study recorded a significant number of participants who had a negative CSF culture. All data were from clinical studies with strict inclusion criteria including expert clinical evaluation. *S.pneumoniae* is a fastidious organism and culture has a low sensitivity, PCR and antigen detection tests were not available. Although our CSF WCC inclusion criteria was >5 cells/mm^3^ in HIV positive participants, the median WCC was significantly higher than this (480 cells/mm^3^ IQR 170–1680). It is unlikely that any participants had a CSF inflammatory response due to seizures alone [39]. All CSF specimens were routinely cultured for Cryptococcus neoformans but not *M.tuberculosis*. Specific TB meningitis (TBM) data from our centre demonstrated a median CSF WCC of only 40 cells/mm^3^ (IQR 0–240) in TBM, 10 fold lower than our study of ABM [40]. All participants with clinically suspected TBM were excluded from the included studies and started on TB treatment. It is unlikely that a significant number of remaining participants in this database had un-diagnosed TBM but cases of TBM cannot completely be excluded.

Some findings contrasted with other studies. Hyponatraemia was associated with mortality, although the numbers were small, contrasting with Dutch data, where no association was seen [19]. Hyperglycaemia was uncommon in our study, although participants with diabetes were excluded from one of the studies [13]. Abnormal glucose levels were not associated with poor outcome, although other studies have shown an association [17]. We were unable to take these observations forward for multivariate analysis due to small numbers of data available. All the studies were undertaken in a profoundly resource-constrained environment where very limited access to laboratory monitoring and intensive care support was available. Further work from our centre will explore these potential associations in the future.

Data heterogeneity limited our analysis. All data were collected prospectively but the lack of uniform data acquisition across the studies and missing data limited our ability to analyse each trend fully. We used random imputation models to test the robustness of our findings, but where the proportion of missing data was higher than collected data those variables could not be included. We were not able to evaluate some of the other indicators that are prognostically significant in other settings. We were unable to stratify our results by CD4 count. A small proportion of adults screened by the included clinical trials died before recruitment, refused consent or were excluded due to study specific criteria, but overall the majority of adults with bacterial meningitis presenting to our centre were enrolled in these studies and the risk of selection bias is low. The consistency of the numbers recruited over each time period and mortality suggests that the confounding effect of time on the analysis is minimal. Despite the introduction of major public health interventions in Malawi over the time period studied the mortality rate from bacterial meningitis remains unchanged.

This study is the largest clinical analysis of bacterial meningitis in adults and adolescents in Africa to date, and the first to investigate the role of clinical presentation and key physiological parameters in outcome. Our findings are likely to be generalisable to much of sub Saharan Africa. Coma, seizures, tachycardia and anaemia all independently predict a poor outcome. Disease severity at presentation is likely to be worsened by delays in presentation to hospital and further in-hospital delays. Early antibiotics and goal directed resuscitation have been shown to reduce mortality significantly in sepsis [41], but have not been tested to date in bacterial meningitis. Further research in Africa is needed urgently to test this approach in resource limited settings in an attempt to improve the in-hospital management of bacterial meningitis and the very high mortality rates.
